# Differing impact of a major biogeographic barrier on genetic structure in two large kangaroos from the monsoon tropics of Northern Australia

**DOI:** 10.1002/ece3.954

**Published:** 2014-01-28

**Authors:** Mark D B Eldridge, Sally Potter, Christopher N Johnson, Euan G Ritchie

**Affiliations:** 1 Australian Museum Research Institute, Australian Museum6 College St, Sydney, New South Wales, 2010, Australia; 2 Research School of Biology, Australian National UniversityActon, Australian Capital Territory, 0200, Australia; 3 School of Marine and Tropical Biology, James Cook UniversityTownsville, Queensland, 4811, Australia; 4 Centre for Integrative Ecology, School of Life and Environmental Sciences, Deakin UniversityBurwood, Victoria, 3125, Australia

**Keywords:** *Macropus antilopinus*, *Macropus robustus*, microsatellites, mitochondrial DNA, Northern Australia, phylogeography, tropical savanna, wallaroo

## Abstract

Tropical savannas cover 20–30% of the world's land surface and exhibit high levels of regional endemism, but the evolutionary histories of their biota remain poorly studied. The most extensive and unmodified tropical savannas occur in Northern Australia, and recent studies suggest this region supports high levels of previously undetected genetic diversity. To examine the importance of barriers to gene flow and the environmental history of Northern Australia in influencing patterns of diversity, we investigated the phylogeography of two closely related, large, vagile macropodid marsupials, the antilopine wallaroo (*Macropus antilopinus; n* = 78), and the common wallaroo (*Macropus robustus; n* = 21). Both species are widespread across the tropical savannas of Australia except across the Carpentarian Barrier (CB) where there is a break in the distribution of *M. antilopinus*. We determined sequence variation in the hypervariable Domain I of the mitochondrial DNA control region and genotyped individuals at 12 polymorphic microsatellite loci to assess the historical and contemporary influence of the CB on these species. Surprisingly, we detected only limited differentiation between the disjunct Northern Territory and Queensland*M. antilopinus* populations. In contrast, the continuously distributed*M. robustus* was highly divergent across the CB. Although unexpected, these contrasting responses appear related to minor differences in species biology. Our results suggest that vicariance may not explain well the phylogeographic patterns in Australia's dynamic monsoonal environments. This is because Quaternary environmental changes in this region have been complex, and diverse individual species’ biologies have resulted in less predictable and idiosyncratic responses.

## Introduction

Despite considerable progress in our understanding of global phylogeography, significant biases remain (Beheregaray 2008). In particular, there has been a strong focus on the Northern Hemisphere and the importance of glacial ice sheets, and elsewhere, on montane fauna and the impact of vicariance events (e.g., the Australian Wet Tropics; Beheregaray 2008). While these studies have advanced our knowledge considerably, other regions and faunal groups require urgent research attention, particularly in light of the current threats posed to biodiversity by global changes to habitat and climate (Brook et al. 2008). A large region that remains poorly studied is Australia's monsoonal tropical savannas (hereafter referred to as Northern Australia).

**Figure 1:**
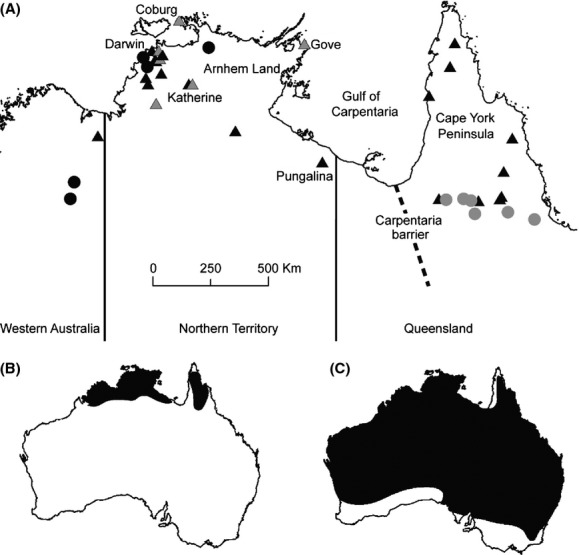
(A) Localities sampled in this study for*Macropus antilopinus* (triangles) and*Macropus robustus* (circles) from the monsoon tropics of Northern Australia. The distribution of the two divergent mtDNA lineages detected within each species is indicated by light and dark shading of the symbols. The distribution of (B)*M. antilopinus* and (C)*M. robustus* in Australia is shown inset below.

Northern Australia is vast, covering nearly one quarter of the continent (∼2,000,000 km^2^), and has high levels of species diversity and endemism (Woinarski et al. 2007). This biome is largely structurally intact compared with other rangelands in the rest of the globe (Bowman et al. 2010). It therefore offers a valuable opportunity to study the phylogeography of species and examine environmental histories, without the potentially confounding effects of recent anthropogenic habitat change and loss. Initial studies from this region indicate a complex history. Species diversity appears to be shaped by periods of past aridification, along with the current influences of the seasonal monsoon, patchy spatial, and temporal distribution of resources, substrates and fire regimes (reviewed in Bowman et al. 2010). Recent phylogeographic studies in Northern Australia have uncovered significant genetic differentiation (e.g., Lee and Edwards 2008; Fujita et al. 2010; Telfer and Eldridge 2010; Toon et al. 2010; Melville et al. 2011; Smith et al. 2011; Potter et al. 2012), with many studies highlighting the importance of the Carpentarian barrier (CB) in shaping species distribution and diversification (e.g., Cardinal and Christidis 2000; Jennings and Edwards 2005; Lee and Edwards 2008; Kearns et al. 2011). The CB (Heatwole 1987), located between Queensland (Qld) and the Northern Territory (NT) at the base of the Gulf of Carpentaria (Fig. 1), is a well-known biogeographic barrier in Northern Australia (Keast 1961; Ford 1987; Bowman et al. 2010). It currently comprises an intrusion of semi-arid grassland separating areas of more mesic savanna woodland to the east on Cape York Peninsula (CYP) and to the west in the “Top End” of the NT (Ritchie et al. 2008; Bowman et al. 2010). During the glacial climatic cycles of the Pleistocene, the CB is likely to have represented a more significant arid barrier than at present (Bowman et al. 2010) and has previously been implicated in intra-and interspecies divergences dating from the mid-Pliocene to the late-Pleistocene (Jennings and Edwards 2005; Toon et al. 2007, 2010; Lee and Edwards 2008; Kearns et al. 2011).

To date, no study has examined the phylogeography of any large-bodied terrestrial (nonvolant) species that span Northern Australia. The antilopine wallaroo (*Macropus antilopinus*) and common wallaroo (*Macropus robustus*), two sympatric species of closely related, large, vagile, macropodid marsupials (Meredith et al. 2008; Van Dyck and Strahan 2008), are ideal candidates to explore the impact of biogeographic processes on patterns of diversification across Northern Australia.

**Figure 2:**
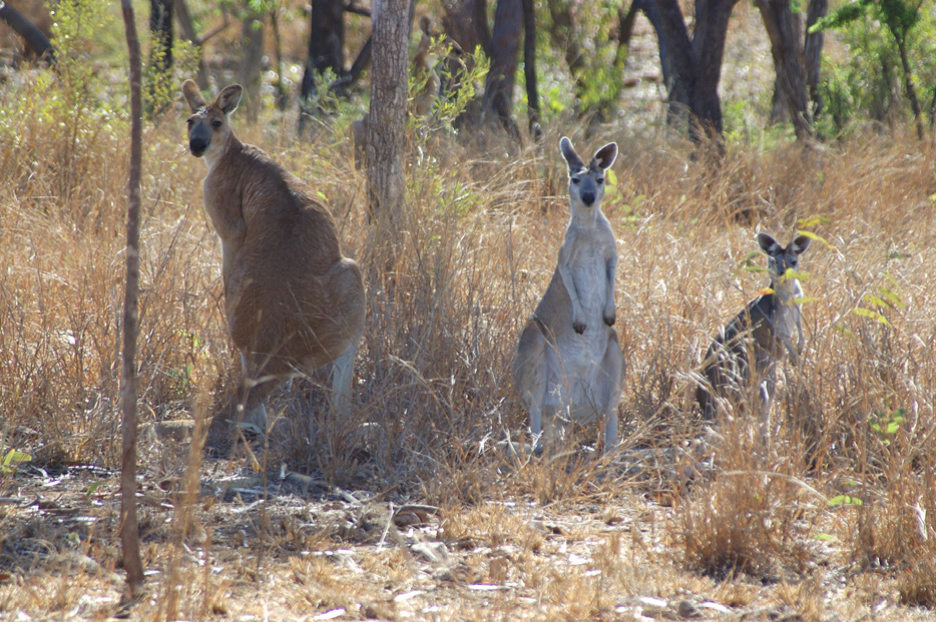
A family group of Antilopine wallaroos (*Macropus antilopinus*) in grassy tropical woodland, Northern Australia. Left to right; adult male, adult female, juvenile female. Photo by Jenny Martin.

The antilopine wallaroo (24–51 kg) is a savanna woodland specialist grazer (Fig. 2) that is endemic to Northern Australia (Van Dyck and Strahan 2008; Ritchie 2010). It is found from the western Kimberley, Western Australia (WA) to CYP with a major disjunction in its distribution coinciding with the CB. Historically, gene flow between the now disjunct populations of antilopine wallaroos in the NT and Qld may have occurred across the Carpentarian Plain, a vast expanse of land (now submerged beneath the Gulf of Carpentaria, Fig. 1) that has periodically connected CYP to the Top End of the NT during periods of low sea level under glacial climates (Chivas et al. 2001; Bowman et al. 2010). Since the most recent marine inundation of this area (∼10 KYA (thousand years ago); Yokoyama et al. 2001) following the last glacial maximum (∼20 KYA), the only connection between*M. antilopinus* populations in Qld and the rest of the species’ range was across the CB.

Across Northern Australia,*M. antilopinus* is sympatric with the common wallaroo (28–60 kg) a widespread generalist browser/grazer that is distributed throughout Australia, including across the CB, and is often associated with rocky areas (Van Dyck and Strahan 2008). Morphological variation within common wallaroos has led to the tentative recognition of several regional subspecies, including*Macropus r. woodwardi* in northwestern and*M. r. robustus* in eastern Australia (Van Dyck and Strahan 2008), although the extent of genetic differentiation amongst regional populations remains unclear (Richardson and Sharman 1976).

This study aimed to use molecular genetic analysis (microsatellites; mitochondrial DNA – mtDNA) of both*M. antilopinus* and*M. robustus* to assess the role of the CB in influencing diversification and evolutionary processes of large nonvolant species in Northern Australia. The differing habitat specificity of these species is likely to reveal the significance of refugia for tropical woodland species on either side of the CB during past aridity cycles, as well as assessing the impact of the CB on contemporary gene flow.*A priori*, we would predict that the disjunct tropical woodland specialist (*M. antilopinus*) would show more pronounced genetic structuring across the CB than the widespread habitat generalist (*M. robustus*). Understanding the impact of the CB in a wide range of taxa will clarify how this vicariant barrier has influenced the current genetic structure of communities across Northern Australia and increase our knowledge of the biogeographical processes shaping patterns of diversity in this region.

## Materials and Methods

### Sample collection and DNA extraction

Samples of ear tissue were collected opportunistically from road-killed*M. antilopinus* and*M. robustus* encountered during field surveys throughout Northern Australia. Additional samples were obtained from wildlife parks, carers, managers, and fellow researchers. In total, samples were obtained from 78 *M. antilopinus* (Qld,*n* = 19; NT,*n* = 55; WA,*n* = 4), 14 sympatric*M. r. woodwardi* from WA (*n* = 6) and NT (*n* = 8) and seven sympatric*M. r. robustus* from northeast Qld. For phylogenetic analyses, three additional*M. r. robustus* from outside the zone of sympatry (northeast Qld,*n* = 2; northeast NSW*n* = 1), two euros (*Macropus r. erubescens*) from WA, one Barrow Island euro (*Macropus r. isabellensis*), and one black-wallaroo (*Macropus bernardus*) were also obtained (see Table S1 for details of collection localities). Samples were preserved in 70–90% ethanol before genomic DNA (gDNA) was extracted using a “salting out” method (Sunnucks and Hales 1996).

### Mitochondrial DNA amplification and screening

We determined DNA sequence variation in the hypervariable Domain I of the mtDNA control region (*CR*) using marsupial-specific primers (Fumagalli et al. 1997) and single-strand conformation polymorphism (SSCP, Sunnucks et al. 2000) as previously described (Browning et al. 2001). We sequenced one to four representatives of each identified haplotype using BigDye termination (Applied Biosystems, Carlsbad, CA) resolved on an AB 3730*xl* capillary sequencer at AGRF (Australian Genome Research Facility), Sydney. Mitochondrial sequences were aligned and edited using Sequencher v4.10.1 (Gene Codes Corporation, Ann Arbor, MI), with haplotypic and nucleotide diversities estimated in Arlequin v3.5.1.2 (Excoffier and Lischer 2010).

### Microsatellite amplification and screening

All sampled*M. antilopinus* and sympatric*M. robustus* from northwest Australia and northeast Queensland were genotyped at 12 polymorphic microsatellite loci. Loci were derived from the following: ten from the tammar wallaby (*Macropus eugenii*):*Me14, Me15, Me16, Me17, Me28*,*T3.1, T15.1, T31.1, T32.1*, and*T46.5* (Taylor and Cooper 1998; Zenger and Cooper 2001a) and two from the eastern grey kangaroo (*Macropus giganteus*):*G16.1* and*G26.4* (Zenger and Cooper 2001b). Microsatellite alleles were amplified from gDNA in three multiplex reactions as previously described (Miller et al. 2011), with fragment analysis conducted on an AB 3730*xl* at the AGRF, Melbourne.

Conformance to Hardy–Weinberg equilibrium (HWE) for each microsatellite locus and linkage disequilibrium (LD) was tested via Genepop v3.1 (Raymond and Rousset 1995) using the Markov chain method with 1000 iterations.*P* values were adjusted using the sequential Bonferroni procedure (Rice 1989). Observed heterozygosity (*H*_o_), expected heterozygosity (*H*_E_), allelic diversity (*A*), and allelic diversity corrected for sample size (*A*_n_*)* were estimated using Fstat v2.9.3 (Goudet 1995). The mean number of rare alleles (*rA*; allele frequency < 0.05) and unique (private) alleles (*uA*) per locus was also calculated. Differences in diversity indices amongst sampled populations were assessed via a Wilcoxon rank sign test (Sokal and Rohlf 1995) using Systat9 (SPSS, Chicago, IL).

### Genetic structure

Pairwise differentiation (mtDNA Φ_ST_; microsatellites*F*_ST_) amongst geographic populations was estimated and tested for significance in Arlequin (mtDNA) and in Fstat (microsatellites) using 1000 permutations. For the mtDNA data, the presence of isolation-by-distance was tested using a Mantel test (Mantel 1967) implemented in zt (Bonnet and Van de Peer 2002) with 10,000 replicates, which assessed the significance of the correlation between the genetic (uncorrected distances estimated in PAUP* version 4b10 (Swofford 2000) and geographic distances of individuals.

To infer population structure from the microsatellite data, we utilized a Bayesian clustering method in Structure 2.3.1 (Pritchard et al. 2000), under the admixture model with α inferred from the data, allele frequencies uncorrelated, and lambda set to 1.0. For the whole data set we tested the number of genetic clusters (*K*) present using values of*K* between one and six. For each*K*, 10 independent replicates were run for 200,000 iterations after a burn-in of 100,000 iterations. The inferred number of populations (*K*) within the sample was deduced using both maximum posterior probability (L[*K*]; Pritchard et al. 2000) and maximum delta log likelihood (ΔK; Evanno et al. 2005) implemented in Structure Harvester v0.6.93 (Earl and vonHoldt 2012). Each identified cluster was subsequently rerun alone to test for additional substructuring within clusters.

### Phylogenetic analyses and Divergence dating

Phylogenetic relationships amongst identified haplotypes were analyzed using maximum-likelihood (ML) in RAxML version 7.0.3 (Stamatakis et al. 2008) and maximum-parsimony (MP) in Paup* with a haplotype from a red kangaroo (*Macropus rufus:* GenBank AJ225163) as the outgroup. For the ML analysis, a GTR+I+G model was estimated with PAUP* v4.0b10; (Swofford 2000) using Modeltest 3.06 (Posada and Crandall 1998), based on the Akaike Information Criterion (AIC). Likelihood analyses were started from a complete random starting tree using the rapid Bootstrap analysis, with 1000 pseudoreplicates and 100 searches per replicate. MP analysis was undertaken using a heuristic search, with random addition (10 replicates), gaps treated as a fifth state, tree bisection and reconnection branch swapping, and 5000 bootstrap replicates.

We also estimated dates of divergence between populations of*M. robustus* and*M. antilopinus* across the CB using fossil calibrations in the program Beast version 1.7.4 (Drummond et al. 2012). Given the paucity of the Australasian fossil record, we included*CR* sequence data from additional cogeners (*M. giganteus* AF443123,*Macropus fuliginosus* EF555400 and*Macropus eugenii*) to provide three fossil calibration points on an extended phylogeny (Meredith et al. 2008). These fossil constraints represent calibration points for nodes deeper in the*Macropus* phylogeny, prior to the divergence of*M. antilopinus* and*M. robustus* (Meredith et al. 2008). These calibrations were based on “hard” and “soft” bound constraints that allowed for fixed constraints associated with fossil information but also variability in timing inclusive of the sequence data. Constraints were placed on three nodes representing monophyletic lineages from Meredith et al. (2008). To estimate most recent common ancestors (MRCAs), monophyly was imposed on the lineages associated with the fossil constraints (e.g., one including all taxa – A; one excluding*M. giganteus* and*M. fuliginosus* – B; and one containing only*M. antilopinus, M. robustus* and*M. eugenii* – C). The three nodes were dated following Meredith et al. (2008 and references therein): 3.62 MYA (million years ago) minimum divergence date for nodes A and B (based on oldest*Macropus* spp. from deposits from Bow and Bluff Downs Local Fauna) with a maximum date of 15.97 MYA (associated with an absence in System C deposits from stratigraphic bounding (Riversleigh)) and a minimum date of 3.4 MYA; for node C, with a maximum of 11.61 MYA (based on absence in Encore Local Fauna stratigraphic bounding). Due to the gaps in the fossil record, a normal prior distribution was implemented to account for any bias in the fossil record. The analysis was run using a relaxed molecular clock, the Yule tree prior, and an uncorrelated lognormal prior and using the GTR+I+G model outlined above. Two independent MCMC chains were run for 10 million generations with sampling every 1000 generations, and a 10% burn-in was removed from the posterior samples. Tracer version 1.5 (Rambaut and Drummond 2007) was used to check that the convergence of parameter estimates (e.g., effective sample size and posterior densities) and log-likelihood values had occurred. Posterior probabilities (pp) were calculated after discarding the first 10% of the sampled trees as burn-in. The coefficient of variation was assessed to see whether rate heterogeneity was present amongst lineages and the covariance parameter was assessed to determine whether there was evidence of autocorrelation of branch rates.

### Historical demography

We tested for current gene flow (migration) across the CB using Migrate-n (Beerli 2009). Migrate-N uses a coalescent approach to estimate demographic processes (e.g., Θ – population size; and*M –* migration rate) where Θ = 4 *N*_e_*μ (N*_e_ is the effective population size and*μ* is the mutation rate per site),*M *= m/*μ* (m is the immigration rate per generation).*Macropus antilopinus* and northern*M. robustus* were separated into populations on either side of the CB, and analyses were run separately for mtDNA and microsatellites using the Bayesian analysis approach (Beerli and Felsenstein 2001; Beerli 2006). Microsatellite analyses used the Brownian mutation model, and the mtDNA analyses used the sequence mutation model.*F*_ST_ estimates were used as starting parameters for estimation of Θ and*M,* and analyses were run starting from a random number seed, a unweighted pair-group mathematic averaging (UPGMA) tree, and excluded missing data. Mutation rates were estimated from the data for microsatellite analyses but constant for the mtDNA analyses. For all analyses, two independent analyses were run, each run with one long chain and four heated chains, sampling every 1000 steps with 400,000 genealogies recorded after a burn-in of 100,000 steps.

Bayesian skyline plots (BSPs) were also constructed to test for historical demographic expansion for*M. antilopinus* and northern*M. robustus* from mtDNA sequences using Beast version 1.7.4. Analyses were run separately for NT/WA and Qld populations, using a strict clock, the HKY model with a proportion of invariant sites, and the γ distribution model based on Modeltest 3.06. A total of 10 million generations were run using five piecewise intervals (three for Qld*M. robustus* analysis) to estimate Ne and a mutation rate of 0.03824 based on the output from the fossil calibrations. Tracer version 1.5 was used to assess convergence based on effective sample sizes, and the BSP analysis was performed using the stepwise constant model.

## Results

### Control region diversity

A total of 48 mtDNA*CR* haplotypes (648 base pairs) were identified within*M. antilopinus,* 16 in Qld (*n* = 19) and 32 in NT/WA (*n* = 57). No haplotypes were shared between the regions (Table S2). Within*M. robustus,* 20*CR* haplotypes were identified, 11 from*M. r. woodwardi* (*n* = 14), six from*M. r. robustus* (*n* = 6), two from*M. r. erubescens* (*n* = 2), and one from*M. r. isabellensis*. Haplotypic diversity was high (∼1.0) amongst the Qld and NT/WA*M. antilopinus* and*M. robustus* populations (Table S2), but nucleotide diversity (*π*) was substantially lower in the Qld*M. antilopinus* population (Table 1). Average sequence divergence (ASD) within the Qld*M. antilopinus* population was 0.8%, compared with 3.4% within the NT/WA population; these were separated by 3.0% ASD. In*M. robustus*, there was 2.6% ASD within the Qld and 4.1% ASD within the WA/NT populations; these were separated by 7.9% ASD. For complete sequences, see GenBank accession numbers KF974367-KF974435.

**Table 1:** Genetic diversity indices (mean ± SE) for four sampled wallaroo populations.

Population	Microsatellite diversity							MtDNA diversity									
	*N*	*A*	*A*_n_	*H*_O_	*H*_E_	*rA*	*uA*	*h* (±SD)	*π* (±SD)
*Ma* – Qld	18.9 ± 0.08	6.7 ± 1.0^2^	5.0 ± 0.6^1^	0.68 ± 0.04^3^	0.68 ± 0.05^3^	2.1 ± 0.6^2^	0.08 ± 0.08^1^	0.98 ± 0.02	0.8 ± 0.5
*Ma* – NT/WA	51.9 ± 0.08	11.5 ± 2.0	6.2 ± 0.7^4^	0.69 ± 0.06	0.73 ± 0.06	6.3 ± 1.8	2.2 ± 0.8	0.97 ± 0.01	3.0 ± 1.5
*Mr –* NT/WA	13.8 ± 0.11	10.3 ± 1.0	8.0 ± 0.7	0.80 ± 0.05	0.81 ± 0.04	4.1 ± 0.8	2.7 ± 0.6	0.96 ± 0.04	3.8 ± 2.0
*Mr –* Qld	9.0 ± 0.00	7.9 ± 0.79^5^,^6^	7.6 ± 0.7	0.76 ± 0.08	0.76 ± 0.07	3.1 ± 0.6^5^	1.3 ± 0.4^6^	1.00 ± 0.13	2.7 ± 1.7

*Ma, Macropus antilopinus; Mr Macropus robustus;* Qld, Queensland; NT, Northern Territory; WA, Western Australia;*N*, sample size;*A,* alleles per locus;*A*_n_, alleles per locus corrected for sample size (*n* = 9);*H*_O_, observed heterozygosity;*H*_E_, expected heterozygosity;*rA*, rare alleles (<0.05) per locus;*uA*, unique alleles per locus;*h,* haplotypic diversity,*π,%* nucleotide diversity.

1 Significantly (*P* < 0.03) lower than all other populations.

2 Significantly (*P* = 0.03) lower than *Ma*-NT/WA and*Mr*-NT.

3 Significantly (*P* < 0.03) lower than *Mr*-NT.

4 Significantly (*P* < 0.04) lower than both *Mr* populations.

5 Significantly (*P* < 0.03) lower than *Ma*-NT/WA.

6 significantly (*P* < 0.04) lower than *Mr*-NT.

### Microsatellite diversity

All microsatellite loci were polymorphic in both*M. antilopinus* and*M. robustus*. All populations were in HWE at all loci (*P *>* *0.05), and there was no consistent evidence of LD (*P* > 0.05). A total of 143 microsatellite alleles were identified in*M. antilopinus* (41% unique) and 158 within*M. robustus* (48% unique; Table S3). Almost all identified*M. antilopinus* alleles 97% (138/143) were found within the NT/WA, while only 56% (80/143) were present in Qld. Within*M. antilopinus*, few of the alleles present in Qld (6%, 5/80) were unique, while 46% (63/138) of alleles were unique to the NT/WA population (Table S3). Despite the small sample of northeast Qld*M. robustus*, 95 alleles were present (17% unique), while 123 alleles were identified in NT/WA (25% unique)*M. robustus* (Table S3).

Levels of genetic diversity were high in NT/WA*M. antilopinus* and*M. robustus* with allelic diversity (*A*) ranging from 10.3 to 11.5 and expected heterozygosity (*H*_E_) from 0.73 to 0.81 (Table 1). Although*H*_E_ was similar in the Qld*M. antilopinus* population (*H*_E_ = 0.68), allelic diversity corrected for sample size (*An*) was significantly lower than all other sampled populations (*P *<* *0.008). The Qld*M. antilopinus* population also had values for*A*,*rA,* and*uA* that were significantly lower than both the NT/WA*M. antilopinus* (*P < *0.02) and*M. robustus* (*P < *0.003) populations (Table 1). The NT/WA*M. antilopinus* population was also significantly lower for*An* than both*M. robustus* populations (*P < *0.04).

### Genetic structure

Genetic differentiation (*F*_ST_ and Φ_ST_) between all four populations was significant, but was substantially lower between the Qld and NT/WA*M. antilopinus* populations (*F*_ST_ = 0.047; Φ_ST_ = 0.270) than between the Qld and NT/WA*M. robustus* populations (*F*_ST_ *=* 0.128; Φ_ST_ = 0.560; Table 2). Significant isolation-by-distance was present within the NT/WA population of*M. antilopinus* (*R* = 0.324,*P *=* *0.002) and*M. robustus* (*R* = 0.513,*P *=* *0.038), as well as within a combined Qld/NT/WA*M. robustus* population (*R* = 0.894,*P *=* *0.0001), but not the Qld*M. antilopinus* (*R* = −0.133,*P *=* *0.099) or*M. robustus* (*R* = −0.128,*P *=* *0.430) populations, nor in a combined Qld/NT/WA*M. antilopinus* population (*R* = 0.072,*P *=* *0.055).

**Table 2:** Genetic differentiation amongst four sampled wallaroo populations.

	*Ma*-Qld	*Ma*-NT/WA	*Mr-*NT/WA	*Mr-*Qld
*Ma*-Qld	–	0.270^1^	0.821^1^	0.893^1^
*Ma*-NT/WA	0.047^1^	–	0.734^1^	0.743^1^
*Mr-*NT/WA	0.211^1^	0.180^1^	–	0.560^1^
*Mr-*Qld	0.215^1^	0.176^1^	0.128^1^	–

*Ma, Macropus antilopinus; Mr Macropus robustus;* Qld, Queensland; NT, Northern Territory; WA, Western Australia.

Pairwise Φ_ST_ values for mitochondrial DNA data above the diagonal and pairwise*F*_ST_ for microsatellite data below the diagonal.

1 Significant (*P* < 0.01) after Bonferroni correction.

**Figure 3:**
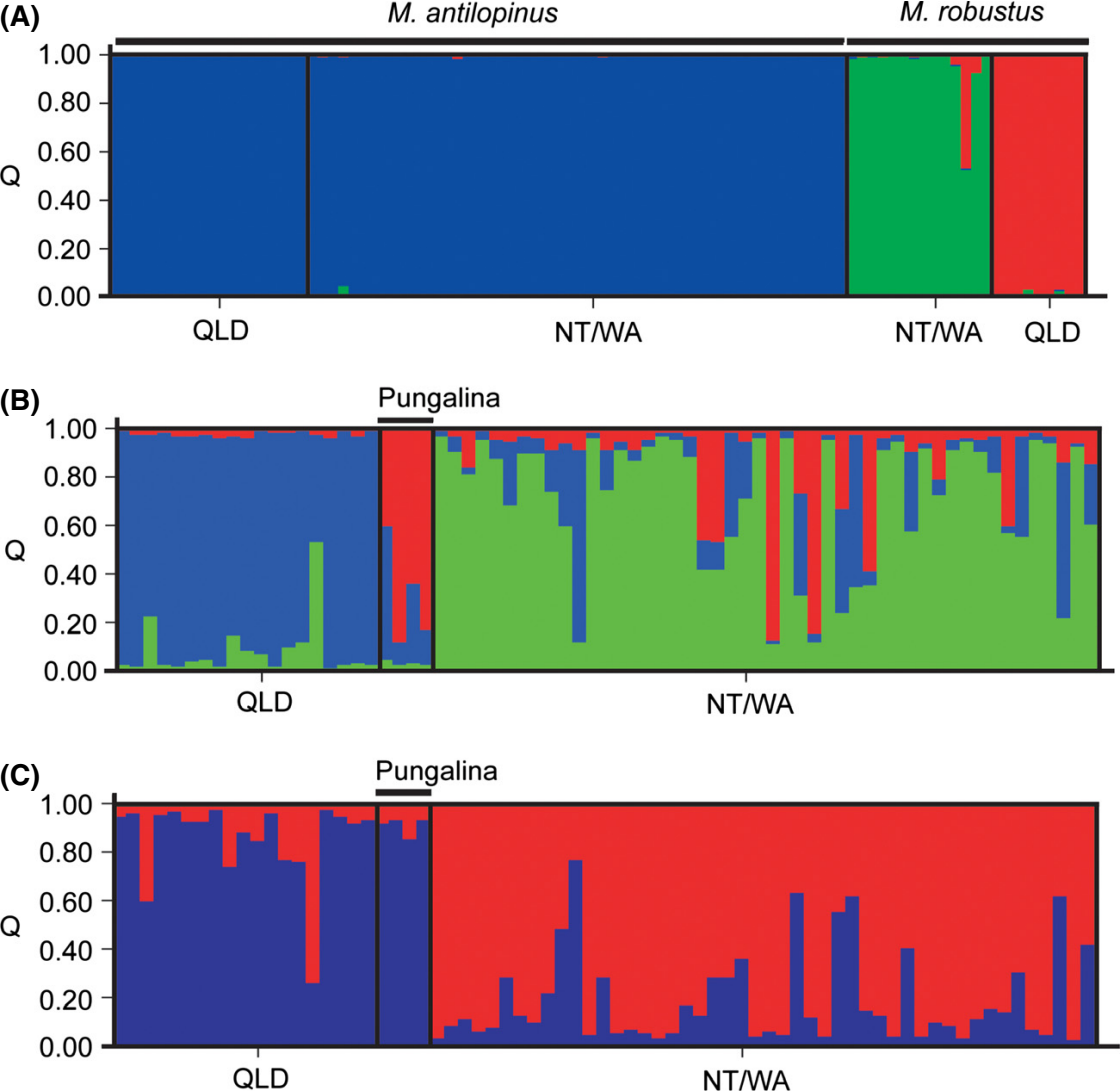
Structure plot showing proportion of inferred ancestry (Q) in the genetic clusters identified within*Macropus antilopinus* and*Macropus robustus* in Northern Australia. For each species, individuals are ordered by decreasing geographic distance from the Carpentarian barrier for the first population and increasing distance for the second. (A) Combined*Macropus antilopinus* and*M. robustus* (*K* = 3). (B)*M. antilopinus* only (*K* = 3). (C)*M. antilopinus* only (*K* = 2).

The Structure analysis indicated that either two (maximum*L*[*K*]) or three (maximum Δ*K*) populations were present in the complete data set (Fig. S1). These inferred populations corresponded to*M. antilopinus* and*M. robustus* when*K *=* *2, or*M. antilopinus* and two*M. robustus* clusters when*K *=* *3 (Fig. 3A). The two*M. robustus* clusters consisted of NT/WA*M. r. woodwardi* and northeast Qld*M. r. robustus*, with some individuals showing admixture (Fig. 3A). These same two*M. robustus* clusters were resolved when the*M. robustus* data were analyzed separately.

Subsequent Structure analyses of the identified*M. antilopinus* cluster revealed additional substructuring with either two (Δ*K*) or three (*L*[*K*]) populations inferred (Fig. S1), but with most individuals showing some admixture (Fig. 3B and C). With*K *=* *2, one cluster consisted mostly of individuals from Qld, but also included all individuals sampled from Pungalina (*n* = 4) in the far eastern NT (see Fig. 1), while the other cluster consisted of individuals from the NT/WA (Fig. 3C). With*K *=* *3, one cluster consisted of all individuals from Qld, another of most individuals from NT/WA, with a third cluster comprised mainly the individuals from Pungalina (Fig. 3B). In both analyses, individuals showing high levels of admixture were not geographically clustered, but widely scattered in the Qld and NT/WA populations (Fig. 3B and C).

### Phylogenetic analyses

**Figure 4:**
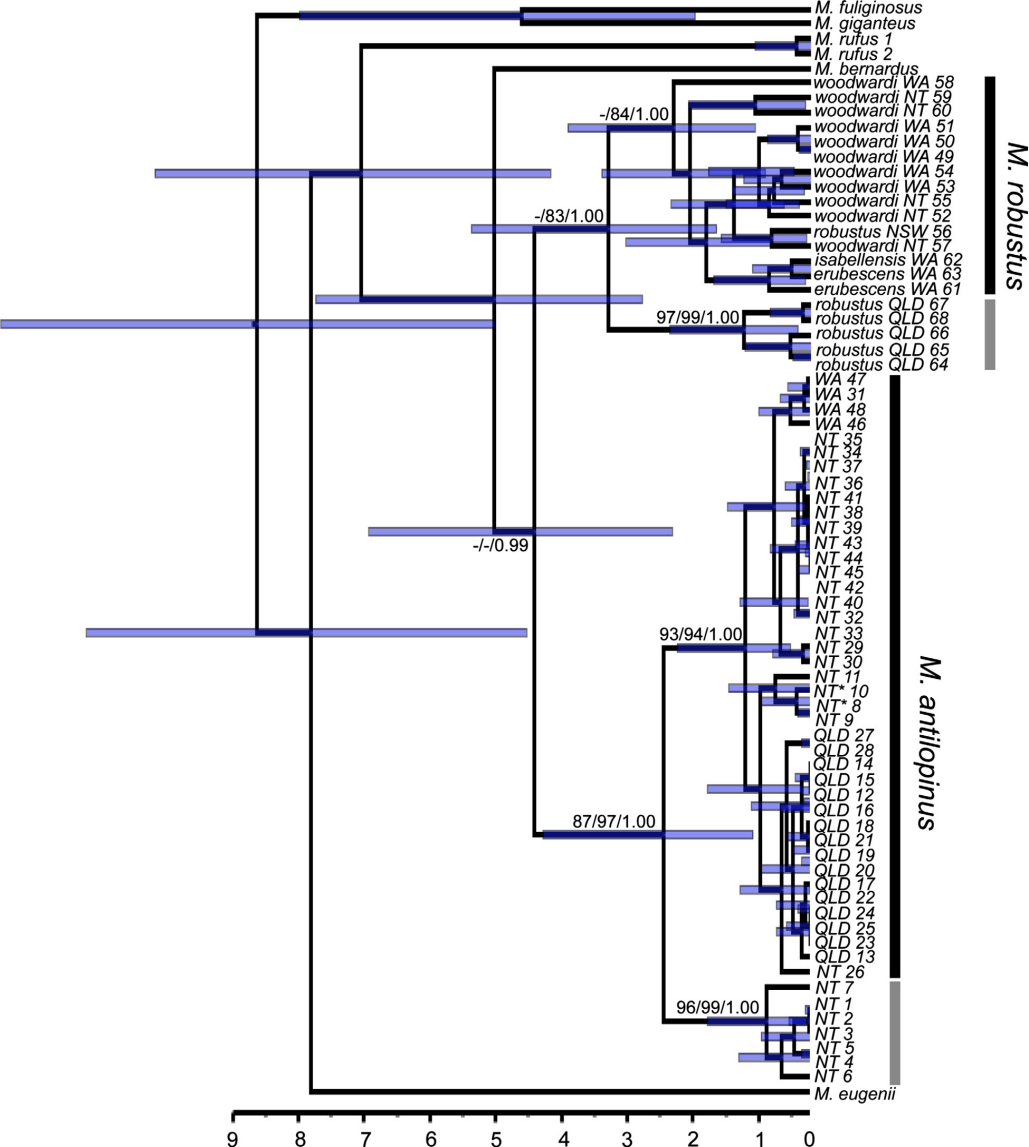
Chronogram inferred from control region (*CR*) haplotypes of*Macropus antilopinus and Macropus robustus*using a relaxed molecular clock in Beast based on the normal prior distribution of fossil calibrations. Values at the nodes represent the mean time since divergence; node bars represent 95% confidence intervals. The scale bar is in millions of years. Individuals from the divergent clade are indicated by the light-shaded bar. Numbers on major branches indicate bootstrap (maximum-likelihood, maximum-parsimony) support or posterior probability (Bayesian) when ≥80% or 0.08. Haplotypes are labeled as in Table S2 and with a broad geographic location.

The results of the MP and ML analysis are not presented separately but are summarized on the Beast tree (Fig. 4). The MP and Bayesian analyses resolved three major lineages amongst the sampled wallaroos corresponding to*M. bernardus*,*M. robustus,* and*M. antilopinus*. The ML tree was similar but differed in failing to resolve a monophyletic*M. robustus* clade. The analyses also differed in their placement of*M. bernardus*. The ML and MP analyses place*M. bernardus* and*M. antilopinus* as sister taxa, while the Bayesian analysis placed*M. robustus* as a sister to*M. antilopinus* (Fig. 4). Amongst wallaroo species, ASD ranged between 12.7% and 14.8%. Within*M. antilopinus*, two well-supported major lineages were resolved in all analyses (Fig. 4), the first comprising a subset of NT haplotypes, the second containing the remaining NT haplotypes, as well as those from Qld and WA (Fig. 4). There was 6.3% ASD between the two*M. antilopinus* clades, 1.6% (ASD) within the NT clade and 1.8% (ASD) within the NT/Qld/WA clade. Two major lineages were also resolved within*M. robustus* (Fig. 4), and these were strongly supported in the MP and Bayesian analyses (Fig. 4). One lineage contained only northeast Qld*M. r. robustus* haplotypes, while the second lineage contained all other*M. robustus* haplotypes (i.e., southeast*M. r. robustus*,*M. r. woodwardi*,*M. r. erubescens*, and*M. r. isabellensis*). Within this second clade,*M. r. woodwardi*and*M. r. robustus* were paraphyletic*,* as were*M. r. erubescens* and*M. r. isabellensis*. There was 7.8% ASD between the two major*M. robustus* clades, 2.6% (ASD) within the northeast Qld*M. r. robustus*clade and 4.5% (ASD) within the widespread*M. robustus* clade. Amongst recognized*M. robustus* subspecies, ASD ranged from 2.3% to 7.3% (mean 5.7%).

### Divergence dating

The covariance results from the Beast analysis spanned zero suggesting no strong evidence of autocorrelation of rates in the phylogeny. In addition, the coefficient of variance did not abut zero suggesting a relaxed molecular clock was appropriate rather than a strict clock for analysis. The two*M. antilopinus* clades were estimated to have diverged 2.453 (1.085–4.279) MYA, while the two*M. robustus* clades were estimated to have diverged 3.278 (1.641–5.372) MYA (Fig. 4). The base of the clade containing only Qld*M. antilopinus* haplotypes was dated to 500 (208–1.110) KYA (Fig. 4).

### Historical demography

Migrate-N detected gene flow across the CB for both*M. antilopinus* and*M. robustus* for the microsatellite and mtDNA data. However, there was a substantial difference in the amount of gene flow across the CB between the two species (Table 3). For the microsatellite data, a higher migration rate was found from the NT/WA into Qld for*M. antilopinus*, almost double on average, whereas for*M. robustus,* the mean migration rates were fairly similar. For the mtDNA data, migration rates were similar in both directions for each species. The unrealistically high values and large confidence intervals reported for*M. robustus* are most likely a consequence of small sample size.

**Table 3:** Gene flow amongst 1 (NT/WA) and 2 (Qld) populations of*Macropus antilopinus* and*Macropus robustus* estimated in Migrate. Mean migration values (immigrants per generation) are*italicized* for microsatellite data and regular for mitochondrial DNA data, and 97.5% highest posterior distribution are given in brackets (e.g., 2.5% and 97.5%).

Species	*M*_2 > 1_	*M*_1 > 2_
*Macropus antilopinus*	*43.4* (*24.0–62.0*)	*71.3* (*50.7–86.7*)
	34.0 (0.0*–*94.8)	31.6 (0.0*–*92.4)
*Macropus robustus*	*8.3* (*0.0–24.7*)	*6.8* (*0.0–23.3*)
	522.9 (0.0*–*579.0)	531.4 (0.0*–*649.0)

**Figure 5:**
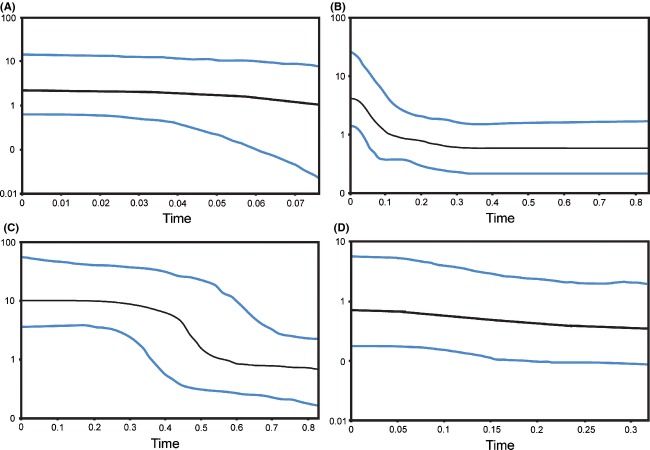
Bayesian skyline plots for*Macropus antilopinus and Macropus robustus* populations. (A)*M. antilopinus*NT/WA. (B)*M. antilopinus* Qld. (C)*M. robustus*NT/WA. (D)*M. robustus* Qld.

The BSP results for*M. antilopinus* indicate a population expansion in the NT/WA (∼200 KYA); however, for Qld, there was no evidence of a change in population size in the last 40 KYA (Fig. 5). For*M. robustus*, BSP results indicate a population expansion for the NT/WA population (∼500 KYA) with the potential for a slight increase in size for the Qld population ∼150 KYA (Fig. 5).

## Discussion

The two sympatric wallaroo species examined in this study showed contrasting patterns of differentiation across the CB, which were the reverse of our*a priori* predictions. The disjunct habitat specialist*M. antilopinus* showed limited differentiation, while the continuously distributed generalist*M. robustus* showed evidence of deep divergence across the CB. Previous phylogeographic studies (mostly of birds) have identified two major patterns across the CB, strong differentiation (e.g., Lee and Edwards 2008; Toon et al. 2010; Kearns et al. 2011) or the apparent absence of an impact (e.g., Kearns et al. 2010; Joseph et al. 2011). While our data for*M. robustus* is consistent with the first pattern,*M. antilopinus* appears to represent a third category, with the CB acting as a partial barrier or a porous filter to gene flow.

### Differential impact of the CB on*Macropus* species

The relatively shallow genetic differentiation identified in this study between the disjunct Qld and NT/WA populations of*M. antilopinus* indicates that the CB is not an absolute historical or contemporary barrier to gene flow. The Qld and NT/WA populations showed limited divergence at mtDNA (Fig. 4) and had comparatively low*F*_ST_ and Φ_ST_ values compared with*M. robustus* and other large macropodids (Table S4). There was also evidence of gene flow across the CB with Migrate-N indicating high levels of gene flow both historically (mtDNA) and more recently (microsatellites; Table 3), as well as individuals from Pungalina (west of the CB) grouping with Qld*M. antilopinus* in some Structure analyses (Fig. 3C). However, there were also indications that the CB does limit gene flow, as evidenced by significant*F*_ST_ and Φ_ST_ vales, the lack of shared mtDNA haplotypes between Qld and NT/WA, and the populations clustering separately in Structure analyses (Fig. 3B).

The low microsatellite and mtDNA nucleotide diversity (Table 2) within the Qld*M. antilopinus* population, the shallow divergence from and lack of reciprocal monophyly with NT/WA populations for mtDNA, suggests a relatively recent (compared with*M. robustus*) movement of*M. antilopinus* from the NT across the CB onto CYP. The BSP showed a population expansion for*M. antilopinus* in the NT/WA about 200 KYA, while the Qld population appears constant from around 40 KYA. The 16 unique*CR* haplotypes found in Qld were restricted to a single*M. antilopinus* clade (Fig. 4) and are closely related (<0.8% ASD), appearing to have diverged ∼500 KYA (208 *K*–1.11 MYA: Fig. 4). In contrast, all*M. antilopinus* haploptypes last shared a common ancestor 2.4 (1.09–4.3) MYA (Fig. 4). In addition, the Migrate-N results support substantially higher rates of gene flow (almost double) across the CB from NT/WA into Qld, than the reverse (43.4 vs. 71.3 average gene flow: Table 3), again suggesting the major direction of movement is west to east.

Although the precise timing of the movement of*M. antilopinus* into northeast Qld is uncertain, it does seem to predate the last glacial maximum (18 KYA), during which the Carpentarian Plain was exposed providing a vast land connection between CYP and the Top End. However, there is no evidence of panmixia between*M. antilopinus* populations across the Carpentarian Plain at this time. This suggests that while paleoecology reconstruction from pollen cores indicates the Carpentarian Plain, when last exposed, supported a tropical savanna (Torgersen et al. 1988; Chivas et al. 2001), it may have provided unsuitable habitat for*M. antilopinus*. Because*M. antilopinus* prefers more dense savanna woodland (Ritchie et al. 2008), the tropical savanna of the Carpentarian Plain may therefore have been more open and grassy, and so unlikely to have facilitated dispersal in*M. antilopinus*. However, this hypothesis requires testing with comparative analyses of other “true” savanna woodland species.

In contrast to*M. antilopinus*, sympatric populations of*M. robustus* show significant differentiation across the CB. The Qld and NT/WA populations showed reciprocal monophyly for mtDNA (Fig. 4), had comparatively high *F*_st_ and Φ_ST_ values compared with*M. antilopinus* and most other large macropodids (Table S4) and clustered separately in the Structure analysis (Fig. 3A). The Migrate-N analysis also detected limited gene flow across the CB, 6.8 (average into Qld) and 8.3 (into NT/WA; Table 3). Although our sampling was limited, the*CR* haplotypes for northeastern Qld*M. r*. *robustus* were highly divergent (7.8%) compared with northwestern (NT/WA)*M. r. woodwardi*. This level of divergence is greater than that amongst all currently recognized*M. robustus* subspecies (mean 5.7%). These data raise some intriguing questions about the interrelationships and evolutionary history of*M. robustus* populations throughout the species’ continental distribution (including many currently recognized subspecies appearing paraphyletic, Fig. 4), and a thorough phylogeographic analysis of*M. robustus* is clearly warranted. Of particular interest would be the pattern of east-west gene flow across southern Australia, beyond the impact of the CB.

Divergence dating suggests that the*M. robustus* populations across the CB last shared a common ancestor 3.278 (1.64–5.37) MYA, which predates most of the divergences reported for other taxa (Jennings and Edwards 2005; Toon et al. 2007, 2010; Lee and Edwards 2008; Kearns et al. 2011), but is similar to the 2.4–5.2 MYA estimated for the savanna woodland honeyeater*Melithreptus albogularis* (Toon et al. 2010). As noted by Toon et al. (2010), these differences highlight major temporal variation in the impact of the CB across taxa.

Thus, our data imply that*M*. *robustus* persisted in refugia on both sides of the CB through the numerous arid cycles of the Pleistocene and that even during more mesic phases, gene flow across the CB was restricted. This finding is contrary to our initial hypothesis, which predicted the widespread generalist (*M. robustus*) to be less impacted by the CB than the disjunct habitat specialist (*M. antilopinus*). Nevertheless, it is intriguing that two closely related sympatric species should have such profoundly different responses to climatic cycling with evidence for both dispersal (*M. antilopinus*) and vicariance (*M. robustus*) across the CB. These radically different responses are challenging to explain but may result from subtle differences in species biology.*M. robustus* is more able to utilize steep and rocky habitats while*M. antilopinus* prefers flat or gently undulating areas (Ritchie et al. 2008). As a consequence,*M. robustus* may have been better able to persist in northeast Qld during extreme aridity cycles due to its superior ability to benefit from the insulating/ameliorating effects of rocky habitat and complex topography on water and nutrient availability and the impact of fire (Burbidge and McKenzie 1989; Mackey et al. 2008). Whereas, because*M. antilopinus* was less able to utilize rocky refugia, it did not persist long term in northeast Qld, but was able to disperse back from the NT when suitable habitat became available. If*M. antilopinus* is typical of other savanna woodland specialists, future research may uncover a similar pattern of recolonization across the CB rather than long-term persistence on CYP through the Pleistocene.

While*M. robustus* was highly structured across the CB, within*M*. *antilopinus,* there was little evidence of genetic structure. In the NT/WA population of*M. antilopinus*,*CR*haplotypes from both major clades (6.3% ASD) were widely dispersed and sometimes co-occurred (e.g., Darwin, Katherine; Fig. 4). While the intermixing of haplotypes from these two clades, whose divergence dates to 2.4 MYA is suggestive of ancient vicariance followed by secondary admixture (Avise et al. 1987), little detail of these hypothesized events can be discerned from our data. Some evidence of regional differentiation was detected amongst contemporary*M. antilopinus* populations. For example, all individuals sampled at Gove (*n* = 4; haplotype NT3), northeast Arnhem Land, and Pungalina (*n* = 4; haplotypes NT8,10) possessed unique haplotypes that were closely related phylogenetically (Fig. 4). This may be a product of our limited sampling, limited female dispersal (as is typical of macropodids (Johnson 1989; Eldridge et al. 2010)), or the effects of isolation-by-distance which was present in the NT/WA*M. antilopinus* population. However, the Pungalina population, which occurs to the west of the CB, was also identified as distinct in the Structure analysis (Fig. 3B), suggesting there may be an additional barrier to dispersal in*M. antilopinus*, west of Pungalina. Our East Kimberley (WA)*M. antilopinus* samples (*n* = 4) also possessed four unique but related haplotypes (WA 31, 46–48; Fig. 4), although more extensive sampling throughout the Kimberley will be necessary before any conclusions can be drawn on about the degree of differentiation between NT and WA*M. antilopinus* populations.

## Conclusion

Biogeographic barriers are of interest to evolutionary biologists due to their expected influence on the historical evolutionary processes of many codistributed taxa. Although the CB is seen as a major vicariance barrier in Northern Australia, our data suggest that it can also act as a porous filter and that dispersal can play a significant role in shaping the distribution and genetic structure of Northern Australian species. This is consistent with evidence of disjunction with subsequent re-expansion for several species now continuously distributed in savanna habitats across this region (e.g., Toon et al. 2007; Lee and Edwards 2008; Toon et al. 2010).

Our results also suggest that the Northern Hemisphere view that patterns of species diversity are largely shaped by periods of glaciation and vicariance events (Hewitt 2004; Shafer et al. 2009), may not explain well the patterns in Australia's dynamic monsoonal environments, where Quaternary environmental changes appear complex (Bowman et al. 2010), and diverse individual species’ biology has resulted in less predictable and idiosyncratic responses. Additional phylogeographic studies of other widely distributed tropical savanna taxa should now be a priority in order to gain a better understanding of the evolutionary history of this globally significant biome.

## Data Accessibility

DNA sequences: Genbank accessions numbers will be added upon acceptance of MS. Microsatellite allele frequencies will be uploaded as online supporting information.

## Supporting Information

**Figure S1.** Inferred number of populations (K) within sampled wallaroos (*Macropus antilopinus and Macropus robustus*) using the maximum posterior probability (L[K] left column: (Pritchard et al. 2000), and maximum delta log-likelihood (ΔK right column: (Evanno et al. 2005)methods implemented in STRUCTURE. **Table S1.** Localities sampled for *Macropus antilopinus* and other wallaroos in this study. **Table S2.** Distribution and frequency of the 68 mitochondrial DNA control region haplotypes identified in sampled *Macropus antilopinus (Ma)* and *Macropus robustus (Mr)* populations. **Table S3.** Observed allele frequencies, at 12 polymorphic microsatellite loci, in sampled *Macropus antilopinus* and *Macropus robustus* populations. **Table S4.** Comparison of population differentiation data for Qld versus NT/WA populations of *Macropus antilopinus* and *Macropus robustus* with published studies for other large macropodids.
