# Supplementary material for: Differing impact of a major biogeographic barrier on genetic structure in two large kangaroos from the monsoon tropics of Northern Australia
Source: Ecol Evol. 2014 Jan 28;4(5):554–67. doi: 10.1002/ece3.954 (PMC4098136; doi:10.1002/ece3.954)

**Supplementary Table 1**. Localities sampled for *Macropus antilopinus* and other wallaroos in this study.

| Taxon | Locality | Latitude  ^o^ ’ S | Longitude  ^o^ ’ E | Sample size |
| --- | --- | --- | --- | --- |
| *M. antilopinus* | ‘Rocky Springs’, Qld | -18° 07’ | 144° 26’ | 3 |
|  | ‘Kendall River’, Qld | -14° 12’ | 141° 37’ | 1 |
|  | Mt Surprise, Qld | -18° 13’ | 144° 22’ | 6 |
|  | ‘Merluna’, Qld | -13° 04’ | 142° 26’ | 2 |
|  | Chillagoe, Qld | -17° 09’ | 144° 31’ | 1 |
|  | ‘Bramwell’, Qld | -12° 09’ | 142° 37’ | 1 |
|  | Georgetown, Qld | -18° 17’ | 143° 32’ | 2 |
|  | Croydon, Qld | -18° 12’ | 142° 14’ | 2 |
|  | Lakeland, Qld | -15° 51’ | 144° 51’ | 1 |
|  | ‘Pungalina’, NT | -16° 47’ | 137° 28’ | 4 |
|  | Litchfield NP, NT | -13° 30’ | 130° 36’ | 1 |
|  | Darwin River, NT | -12° 49’ | 130° 57’ | 2 |
|  | Gove, NT | -12° 11’ | 136° 46’ | 4 |
|  | Daly River, NT | -13° 45’ | 130° 41’ | 4 |
|  | Katherine, NT | -13° 45’ | 132° 16’ | 2 |
|  | 136 km west of Katherine, NT | -14° 30’ | 131° 00’ | 1 |
|  | Darwin, NT | -12° 27’ | 130° 50’ | 6 |
|  | ‘Hodgson River’, NT | -15° 34’ | 134° 05’ | 1 |
|  | Humpty Doo, NT | -12° 37’ | 131° 15’ | 1 |
|  | Batchelor, NT | -13° 05’ | 131° 02’ | 2 |
|  | Berry Springs, NT | -12° 43’ | 131° 00’ | 1 |
|  | Coburg, NT | -11° 16’ | 131° 54’ | 1 |
|  | Territory Wildlife Park, NT | -12° 43’ | 131° 00’ | 8 |
|  | unknown, NT |  |  | 17 |
|  | Kununurra, WA | -15° 46’ | 128° 44’ | 4 |
| *M. r. robustus* | Mt Surprise, Qld | -18° 44’ | 144° 41’ | 1 |
|  | Georgetown, Qld | -17° 48’ | 143° 24’ | 2 |
|  | N of Georgetown | -18° 13’ | 142° 58’ | 2 |
|  | Croydon, Qld | -18° 17’ | 143° 15’ | 1 |
|  | E of Croydon, Qld | -18° 13’ | 142° 58’ | 1 |
|  | Lynd Junction, Qld | -18° 15’ | 142° 17’ | 1 |
|  | Kangaroo Hills, Qld | -19° 01’ | 145° 43’ | 1 |
|  | 7km S Coonabarabran, NSW | -31° 20’ | 149° 18’ | 1 |
| *M. r. woodwardi* | Oenpelli, NT | -12° 20’ | 133° 03’ | 1 |
|  | Territory Wildlife Park, NT | -12° 43’ | 131° 00’ | 5 |
|  | unknown, NT |  |  | 1 |
|  | Batchelor, NT | -13° 05’ | 131° 02’ | 1 |
|  | Hall’s Creek, WA | -18° 13’ | 127° 40’ | 2 |
|  | N of Hall’s Creek, WA | -17° 34’ | 127° 49’ | 4 |
| *M. r. erubescens* | Chapman Valley, WA | -28° 39’ | 144° 42’ | 1 |
|  | Lorna Glen, WA | -30° 10’ | 120° 38’ | 1 |
| *M. r. isabellensis* | Barrow Island, WA | -20° 47’ | 115° 24’ | 1 |
| *M. bernardus* | Arnhem Land, NT | -12° 20’ | 133° 11’ | 1 |

**Supplementary Table 2.**  Distribution and frequency of the 68 mtDNA control region haplotypes identified in sampled *M. antilopinus (Ma)* and *M. robustus (Mr)* populations.

| **MtDNA Haplotype** | **Population** | | | |
| --- | --- | --- | --- | --- |
|  | ***Ma-*Qld** | ***Ma-*NT** | ***Ma-*WA** | ***Mr*** |
| 1 |  | 1, Coburg |  |  |
| 2 |  | 2, Darwin, captive |  |  |
| 3 |  | 4, Gove |  |  |
| 4 |  | 1, w Katherine |  |  |
| 5 |  | 1, Katherine |  |  |
| 6 |  | 1, captive |  |  |
| 7 |  | 1, captive |  |  |
| 8 |  | 2, Pungalina |  |  |
| 9 |  | 1, Daly R |  |  |
| 10 |  | 2, Pungalina |  |  |
| 11 |  | 1, captive |  |  |
| 12 | 1, Merlunna |  |  |  |
| 13 | 1, Merlunna |  |  |  |
| 14 | 1, Georgetown |  |  |  |
| 15 | 1, Bramwell |  |  |  |
| 16 | 1, Rocky Springs |  |  |  |
| 17 | 2, Croydon, Mt Surprise |  |  |  |
| 18 | 1, Mt Surprise |  |  |  |
| 19 | 1, Chillagoe |  |  |  |
| 20 | 1, Lakeland |  |  |  |
| 21 | 1, Mt Surprise |  |  |  |
| 22 | 1, Georgetown |  |  |  |
| 23 | 1, Kendall R |  |  |  |
| 24 | 1, Croydon |  |  |  |
| 25 | 1, Mt Surprise |  |  |  |
| 26 |  | 1, Nutwood |  |  |
| 27 | 2, Rocky Springs |  |  |  |
| 28 | 2, Mt Surprise |  |  |  |
| 29 |  | 3, Daly R, Litchfield, w Katherine, |  |  |
| 30 |  | 1, Darwin |  |  |
| 31 |  |  | 1, Kununurra |  |
| 32 |  | 1, captive |  |  |
| 33 |  | 3, Batchelor, Berry Springs, captive, |  |  |
| 34 |  | 2, Litchfield, captive |  |  |
| 35 |  | 1, Darwin |  |  |
| 36 |  | 3, Darwin, Batchelor, captive |  |  |
| 37 |  | 1, captive |  |  |
| 38 |  | 1, captive |  |  |
| 39 |  | 2, Daly R |  |  |
| 40 |  | 1, captive |  |  |
| 41 |  | 3, Batchelor, captive |  |  |
| 42 |  | 3, Darwin, captive |  |  |
| 43 |  | 7, Humpty Doo, Darwin R, captive, |  |  |
| 44 |  | 2, Darwin, captive |  |  |
| 45 |  | 1, captive |  |  |
| 46 |  |  | 1, Kununurra |  |
| 47 |  |  | 1, Kununurra |  |
| 48 |  |  | 1, Kununurra |  |
| 49 |  |  |  | 1, *Mrw* -Halls Cr |
| 50 |  |  |  | 1, *Mrw* -n Halls Cr |
| 51 |  |  |  | 1, *Mrw* -n Halls Cr |
| 52 |  |  |  | 1, *Mrw* –captive |
| 53 |  |  |  | 1, *Mrw* -n Halls Cr |
| 54 |  |  |  | 1, *Mrw* -n Halls Cr |
| 55 |  |  |  | 3, *Mrw* –captive |
| 56 |  |  |  | 1, *Mrr* –Coonabarabran |
| 57 |  |  |  | 1, *Mrw* –Batchelor |
| 58 |  |  |  | 1, *Mrw* -Halls Cr |
| 59 |  |  |  | 2, *Mrw* –captive |
| 60 |  |  |  | 1, *Mrw* –Oenpelli |
| 61 |  |  |  | 1, *Mre* -Chapman Valley |
| 62 |  |  |  | 1, *Mri* -Barrow Is |
| 63 |  |  |  | 1, *Mre* -Lorna Glen |
| 64 |  |  |  | 1, *Mrr* –Georgetown |
| 65 |  |  |  | 1, *Mrr* -Kangaroo Hills |
| 66 |  |  |  | 1, *Mrr* -Mt Surprise |
| 67 |  |  |  | 1, *Mrr* -n Georgetown |
| 68 |  |  |  | 1, *Mrr* -n Georgetown |

Qld, Queensland; NT, Northern Territory; WA, Western Australia*; Mrw, M. r. woodwardi; Mrr, M. r. robustus; Mre, M. r. erubescens; Mri, M. r. isabellensis*.

**Supplementary Table 3**. Observed allele frequencies, at 12 polymorphic microsatellite loci, in sampled *M. antilopinus* and *M. robustus* populations.

| **Locus** | **Allele (bp)** | ***M. antilopinus* Qld** | ***M. antilopinus* NT** | ***M. robustus* NT/WA** | ***M. robustus* Qld** |
| --- | --- | --- | --- | --- | --- |
| G16.1 | 180 |  |  | 0.036 | 0.222 |
|  | 188 |  |  |  | 0.056 |
|  | 194 | 0.316 | 0.135 |  |  |
|  | 196 | 0.658 | 0.865 | 0.036 | 0.500 |
|  | 198 | 0.026 |  | 0.214 | 0.111 |
|  | 200 |  |  | 0.393 | 0.111 |
|  | 202 |  |  | 0.286 |  |
|  | 204 |  |  | 0.036 |  |
| G26.4 | 195 |  | 0.058 |  |  |
|  | 197 | 0.263 | 0.106 |  |  |
|  | 199 | 0.026 | 0.106 | 0.179 |  |
|  | 201 |  | 0.010 | 0.143 |  |
|  | 203 | 0.474 | 0.394 |  |  |
|  | 205 | 0.158 | 0.154 | 0.071 | 0.167 |
|  | 207 | 0.053 | 0.019 |  |  |
|  | 209 |  | 0.019 |  |  |
|  | 211 |  |  | 0.036 |  |
|  | 215 |  |  | 0.107 |  |
|  | 217 |  |  | 0.071 | 0.056 |
|  | 221 |  |  |  | 0.111 |
|  | 223 |  |  |  | 0.056 |
|  | 225 |  |  | 0.107 | 0.111 |
|  | 227 |  |  | 0.107 | 0.056 |
|  | 231 |  | 0.019 | 0.036 |  |
|  | 233 |  | 0.019 |  | 0.222 |
|  | 235 | 0.026 | 0.038 | 0.036 | 0.111 |
|  | 237 |  | 0.019 |  | 0.056 |
|  | 239 |  | 0.038 | 0.107 | 0.056 |
| Me14 | 198 | 0.342 | 0.010 |  |  |
|  | 200 | 0.184 | 0.240 |  |  |
|  | 202 | 0.026 | 0.106 |  | 0.056 |
|  | 204 | 0.132 | 0.337 |  | 0.278 |
|  | 206 |  | 0.038 | 0.107 | 0.167 |
|  | 208 |  | 0.067 | 0.179 | 0.222 |
|  | 209 | 0.026 | 0.029 |  |  |
|  | 210 |  | 0.048 | 0.286 |  |
|  | 212 |  | 0.096 | 0.179 |  |
|  | 214 | 0.263 | 0.019 | 0.036 |  |
|  | 216 | 0.026 | 0.010 |  |  |
|  | 218 |  |  | 0.036 | 0.056 |
|  | 220 |  |  | 0.071 | 0.111 |
|  | 224 |  |  | 0.071 | 0.056 |
|  | 230 |  |  |  | 0.056 |
|  | 248 |  |  | 0.036 |  |
| Me15 | 247 |  |  |  | 0.111 |
|  | 253 |  | 0.019 |  |  |
|  | 255 |  | 0.038 | 0.036 |  |
|  | 259 |  |  |  | 0.167 |
|  | 263 | 0.639 | 0.500 | 0.036 | 0.056 |
|  | 265 |  |  |  | 0.056 |
|  | 267 |  | 0.087 | 0.071 | 0.167 |
|  | 269 |  | 0.096 |  | 0.111 |
|  | 271 |  |  | 0.036 | 0.111 |
|  | 273 | 0.083 | 0.010 | 0.143 | 0.167 |
|  | 275 |  |  | 0.143 | 0.056 |
|  | 277 |  |  | 0.143 |  |
|  | 279 |  | 0.010 | 0.036 |  |
|  | 281 |  | 0.029 | 0.143 |  |
|  | 283 | 0.028 | 0.048 | 0.107 |  |
|  | 285 | 0.111 | 0.106 |  |  |
|  | 287 | 0.111 | 0.019 |  |  |
|  | 289 | 0.028 | 0.029 | 0.107 |  |
|  | 293 |  | 0.010 |  |  |
| Me16 | 260 | 0.026 | 0.019 |  | 0.056 |
|  | 262 |  |  | 0.071 | 0.111 |
|  | 264 |  | 0.048 |  |  |
|  | 266 |  | 0.048 |  | 0.167 |
|  | 268 |  | 0.202 | 0.250 | 0.056 |
|  | 270 | 0.079 | 0.058 | 0.036 | 0.167 |
|  | 272 | 0.158 |  | 0.071 | 0.056 |
|  | 274 | 0.026 | 0.058 | 0.071 | 0.167 |
|  | 276 |  | 0.154 | 0.143 | 0.111 |
|  | 278 | 0.053 | 0.077 |  |  |
|  | 280 | 0.132 | 0.048 | 0.214 |  |
|  | 282 | 0.421 | 0.077 | 0.071 | 0.111 |
|  | 284 | 0.105 | 0.067 | 0.071 |  |
|  | 286 |  | 0.019 |  |  |
|  | 288 |  | 0.029 |  |  |
|  | 290 |  | 0.019 |  |  |
|  | 292 |  | 0.010 |  |  |
|  | 294 |  | 0.058 |  |  |
|  | 296 |  | 0.010 |  |  |
| Me17 | 143 | 0.421 | 0.404 |  |  |
|  | 147 | 0.053 | 0.231 |  |  |
|  | 149 |  |  |  | 0.222 |
|  | 151 |  |  |  | 0.056 |
|  | 153 | 0.026 | 0.010 |  | 0.056 |
|  | 155 | 0.421 | 0.250 |  |  |
|  | 157 |  | 0.058 |  |  |
|  | 159 | 0.026 | 0.019 |  |  |
|  | 161 |  |  | 0.071 |  |
|  | 167 |  |  | 0.036 | 0.056 |
|  | 169 |  |  | 0.071 | 0.333 |
|  | 171 |  |  | 0.036 | 0.167 |
|  | 173 |  |  | 0.036 | 0.111 |
|  | 175 |  |  | 0.071 |  |
|  | 177 |  |  | 0.071 |  |
|  | 179 | 0.026 | 0.010 |  |  |
|  | 181 | 0.026 |  | 0.036 |  |
|  | 185 |  |  | 0.214 |  |
|  | 187 |  | 0.019 | 0.179 |  |
|  | 189 |  |  | 0.071 |  |
|  | 191 |  |  | 0.071 |  |
|  | 197 |  |  | 0.036 |  |
| Me28 | 158 |  |  | 0.038 |  |
|  | 160 |  |  | 0.077 |  |
|  | 166 |  | 0.029 |  |  |
|  | 168 | 0.105 | 0.019 |  |  |
|  | 170 |  | 0.048 | 0.038 |  |
|  | 172 |  | 0.115 |  |  |
|  | 174 |  | 0.048 |  |  |
|  | 176 |  | 0.019 |  |  |
|  | 178 |  | 0.010 | 0.077 |  |
|  | 180 |  | 0.019 | 0.038 |  |
|  | 182 |  | 0.010 |  | 0.056 |
|  | 184 |  |  | 0.077 | 0.167 |
|  | 186 | 0.026 | 0.067 |  | 0.056 |
|  | 188 |  |  | 0.038 | 0.111 |
|  | 190 | 0.026 |  |  | 0.167 |
|  | 192 | 0.132 | 0.010 |  | 0.111 |
|  | 194 | 0.132 | 0.077 |  |  |
|  | 196 | 0.105 | 0.077 | 0.038 | 0.056 |
|  | 198 | 0.079 | 0.096 | 0.038 |  |
|  | 200 | 0.053 | 0.077 | 0.038 |  |
|  | 202 | 0.079 | 0.038 | 0.038 |  |
|  | 204 | 0.026 |  |  |  |
|  | 206 |  | 0.019 | 0.115 |  |
|  | 208 |  |  | 0.115 | 0.056 |
|  | 210 | 0.026 | 0.029 |  |  |
|  | 212 | 0.026 | 0.019 | 0.038 |  |
|  | 214 | 0.026 | 0.038 |  | 0.111 |
|  | 216 | 0.053 | 0.010 | 0.038 | 0.056 |
|  | 218 |  | 0.019 |  |  |
|  | 220 |  | 0.010 | 0.038 |  |
|  | 222 |  | 0.010 |  |  |
|  | 224 |  | 0.029 | 0.115 |  |
|  | 226 | 0.026 | 0.010 |  | 0.056 |
|  | 228 | 0.026 | 0.010 |  |  |
|  | 232 | 0.053 | 0.010 |  |  |
|  | 234 |  | 0.010 |  |  |
|  | 236 |  | 0.019 |  |  |
| T15.1 | 167 |  | 0.048 |  |  |
|  | 169 | 0.158 | 0.250 |  |  |
|  | 171 | 0.684 | 0.423 |  |  |
|  | 173 | 0.132 | 0.163 |  |  |
|  | 175 |  | 0.010 |  |  |
|  | 177 | 0.026 | 0.010 |  |  |
|  | 179 |  | 0.058 |  | 0.056 |
|  | 181 |  | 0.019 |  |  |
|  | 185 |  | 0.019 |  |  |
|  | 187 |  |  | 0.143 |  |
|  | 189 |  |  | 0.036 | 0.056 |
|  | 195 |  |  | 0.143 |  |
|  | 197 |  |  |  | 0.056 |
|  | 199 |  |  | 0.107 | 0.056 |
|  | 201 |  |  | 0.036 | 0.278 |
|  | 203 |  |  | 0.143 | 0.111 |
|  | 205 |  |  | 0.214 | 0.111 |
|  | 207 |  |  |  | 0.056 |
|  | 209 |  |  | 0.071 |  |
|  | 211 |  |  | 0.071 |  |
|  | 213 |  |  | 0.036 |  |
|  | 217 |  |  |  | 0.056 |
|  | 221 |  |  |  | 0.167 |
| T3.1 | 252 |  | 0.048 | 0.036 |  |
|  | 256 | 0.079 | 0.183 | 0.071 |  |
|  | 260 | 0.421 | 0.279 | 0.071 | 0.063 |
|  | 264 | 0.079 | 0.288 | 0.036 |  |
|  | 268 | 0.079 | 0.048 |  | 0.188 |
|  | 272 | 0.158 | 0.077 |  | 0.063 |
|  | 276 | 0.184 | 0.058 | 0.036 | 0.375 |
|  | 280 |  | 0.019 | 0.286 | 0.063 |
|  | 284 |  |  | 0.143 | 0.188 |
|  | 288 |  |  | 0.107 |  |
|  | 292 |  |  | 0.036 |  |
|  | 296 |  |  | 0.036 |  |
|  | 300 |  |  | 0.036 |  |
|  | 304 |  |  | 0.036 |  |
|  | 308 |  |  | 0.071 | 0.063 |
| T31.1 | null |  |  |  | 1.00 |
|  | 123 |  |  | 0.038 |  |
|  | 127 |  |  | 0.769 |  |
|  | 129 |  |  | 0.192 |  |
|  | 131 |  | 0.029 |  |  |
|  | 133 | 0.763 | 0.712 |  |  |
|  | 135 | 0.026 | 0.192 |  |  |
|  | 137 |  | 0.019 |  |  |
|  | 139 | 0.184 | 0.038 |  |  |
|  | 141 | 0.026 | 0.010 |  |  |
| T32.1 | 169 |  | 0.010 | 0.071 |  |
|  | 171 |  | 0.206 |  |  |
|  | 173 |  |  | 0.071 | 0.111 |
|  | 175 | 0.316 | 0.069 | 0.071 | 0.111 |
|  | 177 | 0.026 | 0.137 | 0.321 | 0.111 |
|  | 179 | 0.237 | 0.196 | 0.071 | 0.056 |
|  | 181 | 0.395 | 0.265 | 0.286 |  |
|  | 183 | 0.026 | 0.029 |  | 0.278 |
|  | 185 |  |  | 0.036 | 0.167 |
|  | 187 |  | 0.010 | 0.036 |  |
|  | 189 |  | 0.078 |  | 0.056 |
|  | 191 |  |  |  | 0.111 |
|  | 193 |  |  | 0.036 |  |
| T46.5 | 170 |  |  | 0.036 |  |
|  | 182 |  |  | 0.036 | 0.111 |
|  | 186 |  | 0.038 |  | 0.111 |
|  | 190 |  | 0.019 |  | 0.056 |
|  | 192 | 0.105 | 0.019 |  |  |
|  | 194 |  | 0.019 |  | 0.222 |
|  | 200 |  |  | 0.107 | 0.056 |
|  | 202 |  | 0.029 |  | 0.111 |
|  | 204 |  |  | 0.107 | 0.111 |
|  | 206 |  | 0.048 |  | 0.056 |
|  | 210 | 0.079 | 0.106 | 0.036 |  |
|  | 212 |  |  | 0.143 | 0.111 |
|  | 214 | 0.132 | 0.212 | 0.179 |  |
|  | 216 |  |  | 0.036 |  |
|  | 218 | 0.263 | 0.288 | 0.143 | 0.056 |
|  | 222 | 0.184 | 0.163 | 0.036 |  |
|  | 224 |  |  | 0.071 |  |
|  | 226 | 0.132 | 0.019 | 0.071 |  |
|  | 230 | 0.105 | 0.038 |  |  |

**Supplementary Table 4**. Comparison of population differentiation data for Qld vs NT/WA populations of *M. antilopinus* and *M. robustus* with published studies for other large macropodids.

|  | ***F_ST_*** | *Φ_ST_* | **Reference** |
| --- | --- | --- | --- |
|  |  |  |  |
| *M. antilopinus* | 0.047 | 0.270 | this study |
| *M. robustus* | 0.270 | 0.560 | this study |
|  |  |  |  |
| *M. giganteus*^1^ | 0.036  (0.004-0.967) | 0.378  (0.040-0.851) | (Zenger *et al.* 2003) |
| *M. fuliginosus*^2^ | 0.063  (0.006-0.217) | 0.583  (0.50-0.99) | (Neaves *et al.* 2009; Neaves *et al.* 2012) |
|  |  |  |  |

^1^ average (and range) for populations sampled 30-1200 km apart

^2^ average (and range) for populations sampled 30-3200 km apart

**Supplementary Figure 1.**

Inferred number of populations (*K*) within sampled wallaroos (*M. antilopinus; M. robustus)* using the maximum posterior probability (*L(K)* left column: (Pritchard *et al.*, 2000), and maximum delta log likelihood (*ΔK* right column: (Evanno *et al.*, 2005) methods implemented in STRUCTURE.

**a)** Combined *M. antilopinus* and *M. robustus* data


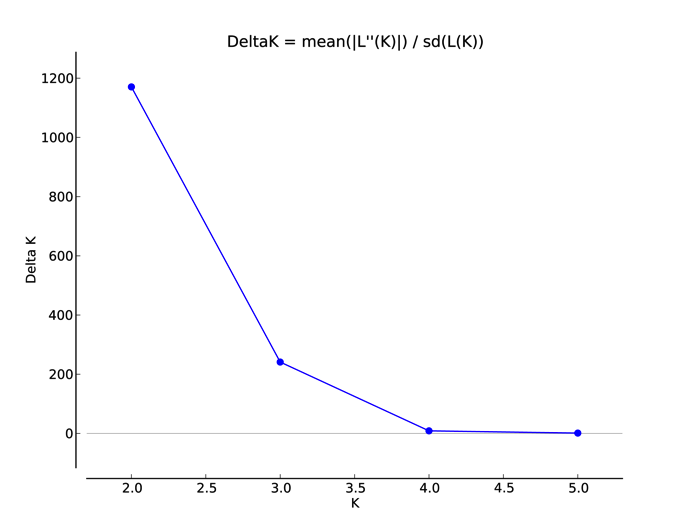

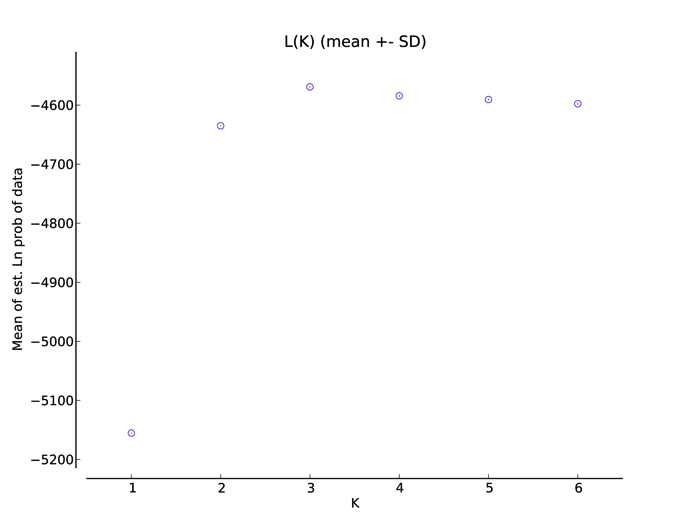


**b)** *M. antilopinus* only


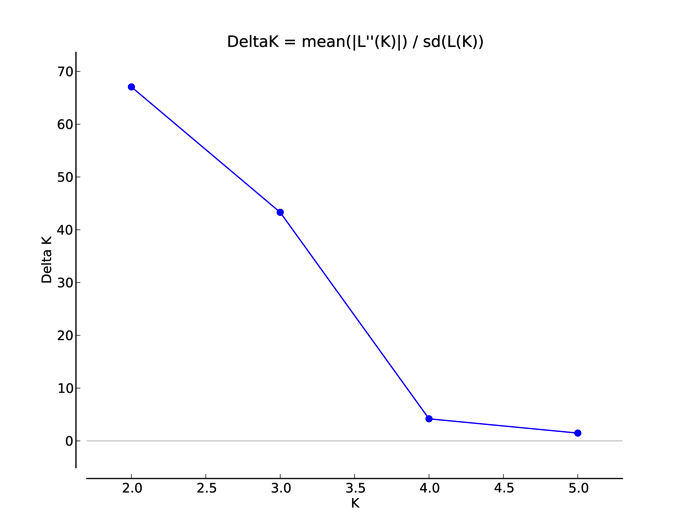

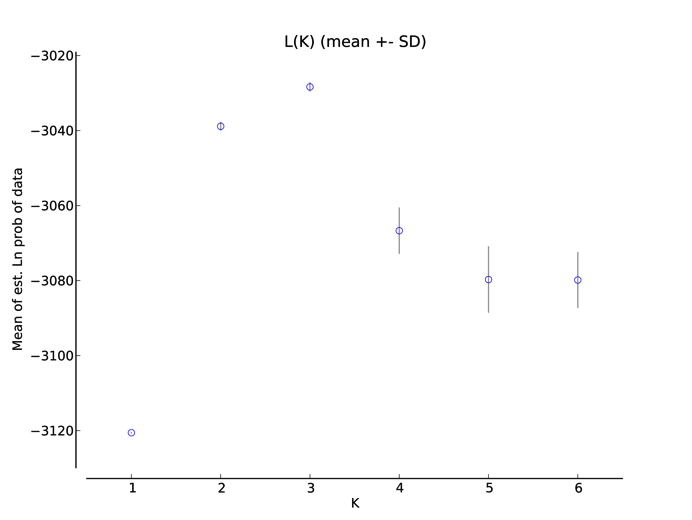


**c)** *M. robustus* only


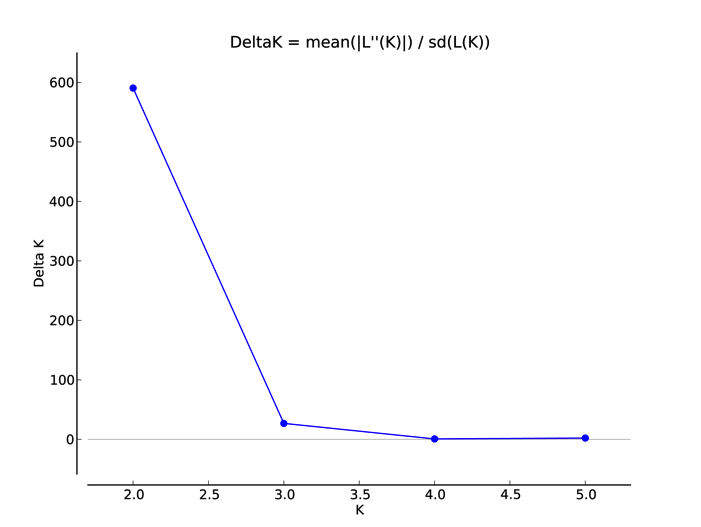

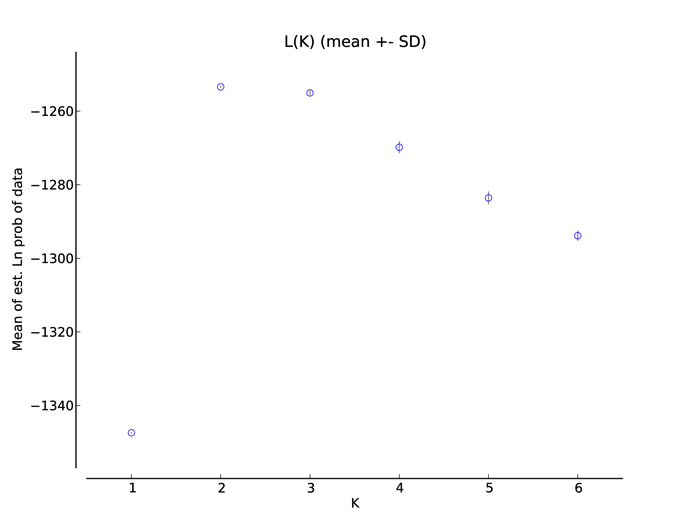

Supplement: Supplementary file 1 — Figure S1. Inferred number of populations (K) within sampled wallaroos (Macropus antilopinus and Macropus robustus) using the maximum posterior probability (L[K] left column: (Pritchard et al. 2000), and maximum delta log-likelihood (ΔK right column: (Evanno et al. 2005)methods implemented in STRUCTURE. Table S1. Localities sampled for Macropus antilopinus and other wallaroos in this study. Table S2. Distribution and frequency of the 68 mitochondrial DNA control region haplotypes identified in sampled Macropus antilopinus (Ma) and Macropus robustus (Mr) populations. Table S3. Observed allele frequencies, at 12 polymorphic microsatellite loci, in sampled Macropus antilopinus and Macropus robustus populations. Table S4. Comparison of population differentiation data for Qld versus NT/WA populations of Macropus antilopinus and Macropus robustus with published studies for other large macropodids. [file ece30004-0554-sd1.docx]
